# Controlling Recombination to Evolve Bacteriophages

**DOI:** 10.3390/cells13070585

**Published:** 2024-03-28

**Authors:** James J. Bull, Holly A. Wichman, Stephen M. Krone, Ian J. Molineux

**Affiliations:** 1 Department of Biological Sciences, University of Idaho, Moscow, ID 83844, USA; hwichman@uidaho.edu; 2 Institute for Modeling Collaboration and Innovation, University of Idaho, Moscow, ID 83844, USA; krone@uidaho.edu; 3 Department of Mathematics and Statistical Science, University of Idaho, Moscow, ID 83844, USA; 4 Institute for Cell and Molecular Biology, Department of Molecular Biosciences, LaMontagne Center for Infectious Diseases, The University of Texas, Austin, TX 78712, USA; molineux@austin.utexas.edu

**Keywords:** phage therapy, computational model, protocol, mathematical model

## Abstract

Recombination among different phages sometimes facilitates their ability to grow on new hosts. Protocols to direct the evolution of phage host range, as might be used in the application of phage therapy, would then benefit from including steps to enable recombination. Applying mathematical and computational models, in addition to experiments using phages T3 and T7, we consider ways that a protocol may influence recombination levels. We first address coinfection, which is the first step to enabling recombination. The multiplicity of infection (MOI, the ratio of phage to cell concentration) is insufficient for predicting (co)infection levels. The force of infection (the rate at which cells are infected) is also critical but is more challenging to measure. Using both a high force of infection and high MOI (>1) for the different phages ensures high levels of coinfection. We also apply a four-genetic-locus model to study protocol effects on recombinant levels. Recombinants accumulate over multiple generations of phage growth, less so if one phage outgrows the other. Supplementing the phage pool with the low-fitness phage recovers some of this ‘lost’ recombination. Overall, fine tuning of phage recombination rates will not be practical with wild phages, but qualitative enhancement can be attained with some basic procedures.

## 1. Introduction

Phage therapy is the use of bacteriophages to treat infections [1,2,3,4,5,6,7,8,9,10,11,12,13,14,15,16,17]. One of the purported benefits of phages over antibiotics is that individual phages typically only have a narrow set of hosts that they can infect, thus limiting the degree to which a microbiome is disrupted by treatment. However, the limited host ranges of phages creates its own problem in identifying phages that will attack and kill the specific bacteria in a patient’s infection [15,18,19,20]. Libraries of phages are typically assembled from newly isolated or long-term collections of natural phages, and large libraries can expedite the discovery and preparation of phages for treating specific infections. Yet these libraries, although extensive, may sometimes be too shallow to include phages against the bacteria causing infections. Furthermore, bacteria may quickly evolve resistance to phages during treatment, and these resistant bacteria can then prove untreatable due to a lack of suitable phages [15,19,20].

A possible solution to these problems is the ‘directed evolution’ of phages to grow on new hosts. In basic outline, directed evolution merely involves exposing phages to the desired bacterial targets. When it comes to actually implementing directed evolution, there are many possible protocols [21,22,23,24,25,26,27,28]. However, one theme emerging in the directed evolution of phage host ranges is that recombination among different phages may greatly accelerate the evolution of growth on new hosts [22].

Recombination has been a focus of research for over a century, first in eukaryotes [29] and later in microbes [30], even being a tool underlying the ‘directed evolution’ of molecules [31,32,33]. However, compared to most contexts, the control of recombination is different in phages. Although phage replication may involve recombination between progeny genomes, phages do not rely on recombination with different phages for their reproduction. Rather, recombination between different phages is likely an accidental outcome of two coinfecting genomes sharing regions of nucleotide identity so that the researcher wishing to incorporate phage recombination must apply a protocol that specifically enables it. Our focus here is to explore how best to control recombination in a protocol and, indeed, the extent to which it is feasible to control recombination. In addressing this question, our main approach is to apply mathematical models of phage-bacterial coinfection and recombination, which we then analyze numerically. We offer some preliminary empirical studies to illustrate the utility and challenges of adhering to models.

## 2. Results

### 2.1. Perspective

Most phage biologists have a practical sense of how to recombine phages: grow two (or more) phages in a culture with a host that is permissive for the phages. Recombinants can, preferably, then be detected by plaque formation on a host that is non-permissive for both parents. This paper will consider (i) whether it is practical to refine this protocol to enhance recombination and (ii) how this approach may result in poor outcomes. For example, is attaining a high ratio of phages to cells (a high multiplicity of infection or MOI) sufficient for enforcing high numbers of recombinants? How sensitive are recombination levels to the abundance of the different phages? Modeling enables us to answer these questions, but then the challenge becomes one of empirically validating and implementing the assumptions underlying the model. Nevertheless, models serve the important role of informing us of the consequences of different protocols and of the different properties of phage life histories. If the models indicate that precise measurements of phage properties must be measured before it is possible to accurately control recombination levels, and if these measurements are impractical with uncharacterized (e.g., wild) phages, then the researcher may want to use protocols based on practicality rather than optimality.

### 2.2. Basics of Modeling Phage Infection

The initial step in controlling phage recombination lies with infection, i.e., coinfecting cells with different phages. Infection processes have been described for almost a century, at least for the common laboratory environment of liquid culture [34,35,36,37]. Phage infection of a bacterial culture is routinely modeled by the laws of mass action, in which bacteria and phage collide and phage infect at a rate that is the product of their two densities and a coefficient (*kPB*, where *B* is bacterial density, *P* is phage density, and *k* is the adsorption rate constant, with units of time commonly in minutes and volume in milliliters (mL)). The adsorption rate constant is a composite measure of the rate at which a cell encounters a phage and the encounter results in an infection [38]. In practice, phage densities in a culture can reach 10^11^/mL, bacterial densities can reach 10^9^/mL, and the adsorption rate coefficient is bounded on the high end by ~10^−8^ due to size and volume constraints [36]. If *k* falls to 10^−11^ or lower, infection may be too slow for phages to affect bacterial densities significantly, but it may be high enough to maintain phages at high density despite their ongoing loss or death [39]. Standard models of infection can accommodate densities that are orders of magnitude higher or lower than can actually be maintained in practice, but extreme densities are not relevant to recombination protocols.

### 2.3. Coinfection

In the absence of genetic engineering manipulations (e.g., [27,31,40,41]), bacteriophage recombination requires coinfection. Coinfection does not guarantee recombination, as the phages may have incompatible life cycles [36] or differ too much in sequence. Nonetheless, coinfection is the step most easily manipulated by the researcher that has a direct effect on recombination. Often the goal will be to maximize coinfections, as individual recombinants may be rare but will increase with coinfection levels. There are, however, potentially subtle questions in choosing an appropriate measure of coinfection to maximize the goal of recombination. For example, is it better to maximize the total number of coinfections or maximize the fraction of coinfections among all infections? Is a high multiplicity of infection (MOI) of the different phages an assurance of high levels of coinfection? If introducing low densities of phages where recombination is desired, is an eventual high MOI of all phages ensured?

We use numerical analysis of a mathematical model of phage-bacterial infection to answer some of these questions. Our initial model tracks the densities of two phage types and a single bacterium in a short interval of time to represent a single cycle of growth (50 min); bacteria are assumed not to grow, and the model counts coinfections (cells infected by both phages), single infections (cells infected by only one type of phage), and uninfected bacteria. The only constraint we impose on coinfections is that infection by the second phage type must occur within an average of 2 min of infection by the first phage type. This limit is inspired biologically, in that the second phage must infect before the first phage has become so dominant in the cell metabolism that the second phage cannot replicate its genome [36], but the model is easily changed to allow a shorter or longer interval. We impose no limit to the number of times a cell may adsorb phages, so all cells (infected or not) act as phage sinks throughout the process. We limit the calculations to coinfections rather than including progeny phage following infection/coinfection, as the progeny phage will depend on burst sizes of single infections and coinfections. Obviously, the chosen parameters in this initial model can be varied, and the model can also be made more complex to represent specific instances. The model could even be made more general, but these trials serve our goal of evaluating conditions where recombination between parental phages is desirable.

Our calculations consider only a single round of infection/co-infection. When less than 50% of cells are infected, there will be a substantial subsequent round of infection that may contribute to coinfections. The level of coinfection in these subsequent rounds may be difficult to control and quantify and thus difficult to model. In this case, the results here may be of limited value, but, in any case, the investigator will often be able to raise the initial levels of infection.

**Figure 1:**
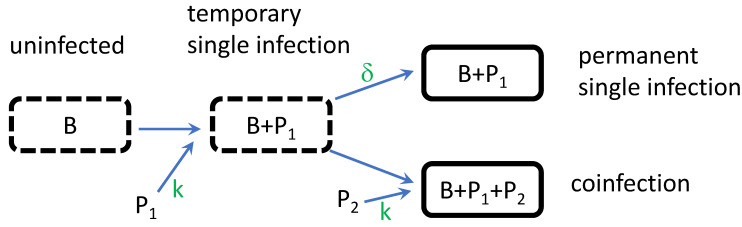
Model flow. Bacteria are indicated by borders; dashed borders indicate that the state can change, solid is permanent (because the model does not extend to lysis of infected cells). Letters inside the borders indicate the contents. *B* = bacteria, *P_i_* indicates phage type *i*, where *i* = 1, 2. Uninfected bacteria can only become singly infected at a given instant. Singly infected bacteria can move into a state of permanent single infection (rate *δ*) or be subject to coinfection. The adsorption rate constant (*k*) influences the rate at which bacteria become infected, independent of whether they were previously infected. These assumptions are for modeling convenience but should be suitable approximations for many empirical systems.

The model uses ordinary differential equations of the densities. Only two parameters are minimally required: the adsorption rate constant, assumed to be equal for the two phages (*k* = 10^−9^ mL/min in all trials for both phages, and regardless of any prior infection of the cell), and the rate at which singly infected cells become ineligible for becoming coinfected (δ = 0.5, hence an average of 2 min). Assigning different adsorption rate constants to each phage would simply add a third parameter. Figure 1 shows the key steps in the model.

The ordinary differential equations in (1) describe the full process, with notations provided in Table 1; a prime (′) indicates a derivative with respect to time. The equations are not essential to understanding the paper, but they are useful for understanding some specific points below.

$$B^{\prime }=B\left(r-kP_{1}-kP_{2}\right)$$

(1) $$P_{1}^{\prime }=-k\;P_{1}\;\left(B+X_{1T}+X_{2T}+X_{1P}+X_{2P}+X_{12}\right)$$

$$P_{2}^{\prime }=-k\;P_{2}\;\left(B+X_{1T}+X_{2T}+X_{1P}+X_{2P}+X_{12}\right)$$

$$X_{1T}^{\prime }=k\;P_{1}\;B-k\;X_{1T}\;P_{2}-\delta \;X_{1T}$$

$$X_{2T}^{\prime }=k\;P_{2}\;B-k\;X_{2T}\;P_{1}-\delta \;X_{2T}$$

$$X_{1P}^{\prime }=\delta \;X_{1T}$$

$$X_{2P}^{\prime }=\delta \;X_{2T}$$

$$X_{12}^{\prime }=k\;X_{1T}\;P_{2}+k\;X_{2T}\;P_{1}$$

For the adsorption rate used, 50 min is sufficient for more than 99% of the phage to be adsorbed when using 10^8^ cells, 40% to be adsorbed by 10^7^ cells, and 5% to be adsorbed by 10^6^ cells. Of greater interest, however, is the fraction of cells that are eventually infected and coinfected, as addressed below.

The model enables calculation of various statistics that may be relevant when designing protocols. The chief purpose here is to illustrate qualitative behaviors and to identify properties of coinfection whose importance may not be obvious. Results are presented as separate heat maps, each for a given initial density of bacteria (10^6^, 10^7^, 10^8^ per mL) across a range of phage densities.

#### 2.3.1. Numbers of Coinfections: MOI vs. FOI

**Figure 2:**
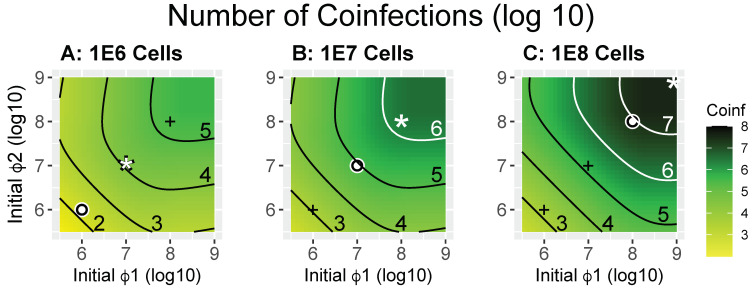
Heatmaps showing the numbers of coinfected cells during 50 min of exposure to phages at the initial phage density indicated (calculated as X_12_(50) in Equation (1)). The *X* and *Y* axes indicate the (log) initial density of Phages 1 and 2 (ϕ1, ϕ2). Panels (**A**–**C**) differ only in the number of bacteria introduced, from 10^6^ to 10^7^ to 10^8^ (as given by the panel titles). Each contour is labeled with the log_10_ of the density of coinfections attained. The white dot in each panel indicates the point at which both phage densities equal bacterial densities (MOI = 1 for both phages), the white star indicates an MOI = 2, and the ‘+’ marks in each panel are merely reference points to the white dots in the other two panels. It is easily seen that higher numbers of coinfections are achieved with higher cell densities, although coinfection densities in panels (**B**,**C**) are nearly the same in the lower left halves of the panels when phage densities are low. Contour lines are black or white (merely to enhance contrast with the background). The code used to generate the numbers used in these figures is provided in Supplemental File S1_coinfection.c.

Possibly the most useful measure relevant to maximizing opportunities for recombination is the actual number of coinfections. When comparing the numerical results (Figure 2), it is immediately clear that the commonly used MOI is a poor indicator of coinfection numbers [10] (for an in-depth analysis of problems with MOI see [42]). In each panel, the white circle is the point at which MOI = 1 for both phages, yet coinfections at MOI = 1 vary by five orders of magnitude across the three bacterial densities even though bacterial densities differ by only two orders of magnitude. The panels also show outcomes for MOI = 2 (white star). The outcomes for MOI = 2 differ by four orders of magnitude across the three cases. The outcomes for MOI = 0.1 can also be inferred.

It is easy to understand that MOI = 1 for both phages does not mean that every cell will be infected. For a single phage, an MOI = 1 results in 63% of the cells eventually becoming infected in an unlimited time (the Poisson probability, see derivation in Appendix A). For coinfection by two phages, each at an MOI = 1, only ~40% (63% × 63%) of cells will be doubly infected in an unlimited time. However, by these calculations, the distribution of (co)infected cells should be more or less equal in all bacterial densities at the same MOI, so they cannot explain the data in Figure 2. The poor predictive power of MOI across the panels arises because the MOI does not indicate the rate at which cells become infected. A low rate means that fewer cells will be infected by both phages within the time during which a coinfection is permitted (2 min in our initial application of the model).

We suggest that a better grasp of these behaviors can be achieved by considering the ‘force of infection’ (FOI) as well as the MOI (we use ‘Force of Infection’ here because it is a term used in epidemiology to indicate the rate at which individuals are infected by a pathogen). Here, the FOI is the product of phage density and the adsorption rate constant, kP. It has units of /min and indicates how fast infections occur at a specified initial phage density, thus describing the initial rate of infection. The initial rate is critical to coinfection because of the constraint imposed by the parameter imposed that two infections must occur within 2 min of each other for recombination to occur (2 min = 1/*δ*). However, whereas the MOI can be obtained from phage and cell concentrations alone, the FOI requires knowing phage concentrations (not cells) in addition to requiring knowledge of a further parameter, the adsorption rate constant. The adsorption rate constant is a per-phage measure of how quickly a phage will infect a bacterium in liquid, and it is routinely measured with standard assays (see [38] for a review of bacteriophage adsorption).

When there is a fixed adsorption rate constant (*k*), there is a 1:1 correspondence between the FOI and phage density (Table 2). For the trials of Figure 2, in which *k* = 10^−9^, FOI = 1 applies at a value of 9 on each axis because 10^9^ × 10^−9^ = 1. Likewise, FOI = 0.1 applies at a value of 8, and so on (Table 2). Thus, the axes can equally be labeled with values of 9, 8, …, to represent (log) phage density or with 1, 0.1, …, to represent FOI. Note that the initial FOI cannot be used by itself to calculate long term coinfection levels. First, it only reflects the initial rate, just as the phage density is only the initial density. Second, it does not account for cell density and, thus, whether each cell is likely to be infected many times or not at all. However, the FOI does provide a comparative basis for understanding why the MOI = 1 condition at different bacterial cell densities leads to strikingly different coinfection levels, i.e., the MOI = 1 condition applies at an FOI level of 0.001 in Panel A, a level of 0.01 in Panel B and a level of 0.1 in Panel C, spanning the same two orders of magnitude as bacterial cell densities.

Comparing maximum coinfections (the upper right corner of each panel in Figure 2), there is an approximate 10-fold increase in number of coinfections with a 10-fold increase in cell density as one moves from Panel A to B to C. FOI = 1 applies to the upper right corners. This shows the utility of a high FOI, because coinfections increase proportionally with cell density. Necessarily, this pattern would not hold when cell density is increased indefinitely while holding the FOI constant, as there are fewer and fewer phages per cell; thus, the MOI is also relevant.

Comparison of the expectations from each bacterial cell density panel reveals that, at all phage densities (hence at all FOI levels), higher cell densities lead either to higher coinfection numbers or to no substantial change. It might seem that low MOI levels would disproportionately hamper coinfections, but the coinfection level at the lower left “+” in Panel C—which is an MOI of 0.01—is actually greater than the coinfection level in Panel A, where the MOI is 1.0. This suggests a gestalt strategy of including more cells in the culture to enhance coinfections. However, it should again be remembered that these numbers are for the first round of infection, and the user may have no control over subsequent rounds.

The parameterization employed assumes equal adsorption rates for the two phages. However, the fact that phage densities are always multiplied by adsorption rate constants in the model means that the same graphs can be used to encompass adsorption rate differences between coinfecting phages. Thus, if the adsorption rate constant for Phage 1 is 10^−9^ and that for Phage 2 is 10^−8^, the graph coordinate (8, 9) in Figure 2 would correspond to the level of coinfections when each phage was introduced at 10^8^—the phage density is adjusted to reflect the increase or decrease in adsorption rate from 10^−9^. Likewise, if phage density was replaced with FOI on the Figure 2 axes, a single FOI value is compatible with many combinations of phage density and adsorption rate constant.

#### 2.3.2. Coinfections Relative to All Infections

**Figure 3:**
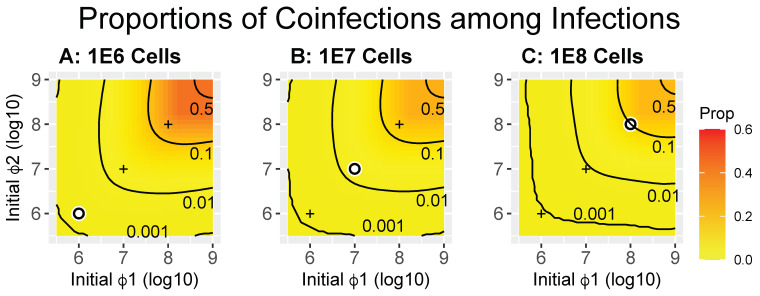
Heatmaps of coinfected cells as a proportion of all infected cells (calculated as X_12_/(X_12_ + X_1T_ + X_2T_ + X_1P_ + X_2P_) from Equation (1) at time = 50). Contours are labeled by the value of the proportion. Trials are the same as in Figure 2. In contrast to Figure 2, there is only a small difference observed for this measure. Panels (**A**–**C**) differ only in the number of bacteria introduced, as given by the titles. The white dot in each panel indicates the point at which both phage densities equal bacterial densities (MOI = 1); for convenience of comparison, the ‘+’ marks in each panel are merely reference points to the white dots in the other two panels.

If there is an upper limit on the total number of phages that can be maintained at some stage of a protocol, it may be more desirable to achieve high levels of coinfection on a per-infected cell basis than to achieve high total numbers of coinfections. Figure 3 shows the proportions of coinfections per infection for the same trials used in Figure 2. Differences across the panel are subtle and not likely to be of practical importance, and whatever one does to increase numbers of coinfections, the proportions of coinfections per infection will not be much affected. There is a mild tendency for coinfections per infection to be greater with fewer cells, with contours shifting slightly away from the upper right. Under realistic conditions, the magnitude of the effect is not intuitive but can be understood in the extreme: when cells vastly outnumber phages, most infections will be single.

#### 2.3.3. Proportions of Cells Infected

The disparity between MOI and the level of coinfection noted above stems substantially from the rate of infection varying across conditions (i.e., from the FOI). At low FOI, hosts are infected infrequently. If a protocol leads to a substantial delay in cells becoming infected, then there will be multiple rounds of infections and bursts before most cells are infected. Indeed, the standard process for generating a cleared lysate relies on multiple generations of phage growth from a small phage inoculum that eventually overwhelms the cells. When starting with low phage densities, growth will lead to high levels of infections. However, attaining high levels of coinfection requires that different phage types increase in concentration at the same rate, as when they have similar latent periods and burst sizes. Such growth equivalence of different phages cannot be assumed when developing general protocols. Coinfection is most easily assured by adding high levels of each phage to the cells so that most cells are infected in the initial round.

**Figure 4:**
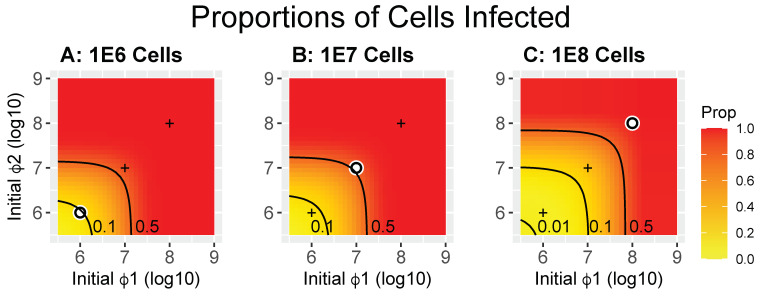
Heatmaps showing the proportions of cells infected by at least one phage for the same trials shown in Figure 2 and Figure 3 (calculated as (X_12_ + X_1T_ + X_2T_ + X_1P_ + X_2P_)/B(0) in Equation (1), where the X_ij_ are at time = 50). These proportions apply to cells infected by the added phages—the first round of infection. Any uninfected cells are available to be infected after the initial infection releases progeny. The white dot in each panel indicates the point at which both phage densities equal bacterial densities (MOI = 1); for convenience of comparison, the ‘+’ marks in each panel are merely reference points to the white dots in the other two panels.

A sense of the problem of how best to infect most cells with two different phages in one round is indicated in Figure 4, again using the same trials as in Figure 2 and Figure 3. The uppermost contour in all panels is 50% coinfection, a value that seems both reasonable and practical. There are two ways to look at these results, and they lead to opposite interpretations. One perspective is that, when holding phage densities constant, higher cell densities lead to lower coinfection rates; this is obvious when cell densities exceed those of the phage and is illustrated by the contour lines moving to the upper right as cell densities increase. A different perspective is obtained when viewing the results relative to the point at which MOI = 1, as lower cell densities result in a lower fraction of cells being coinfected. These two perspectives merely differ in their reference points, one uses MOI = 1 (i.e., both cell and phage density vary) as the reference while the other uses phage density alone. Both perspectives are valid and illuminate the complexity of deciding how best to design an appropriate protocol.

#### 2.3.4. Experimental Measures

To obtain a sense of the ease or difficulty of deliberately controlling coinfection levels, a simple experimental system was developed using two phages with complementing deletions. Historically, mutants of the essential genes of phage T7 were able to grow only when the cell was coinfected by two different mutants that complement each other [43]. The advent of cloning subsequently allowed the host to express only the mutant gene in *trans*. When using two T7 phages with different genetic defects, coinfection of a non-complementing host allows for recombination, such that the progeny contains viable phages, which necessarily form plaques. The appearance of plaques on non-complementing hosts when plating pairs of deletion phages is thus a minimal measure of coinfection.

We used T7 ∆1 (RNA polymerase) and ∆16 (an internal virion protein). Viable particles of each are obtained by growth on a complementing host. They can infect non-complementing cells, but infection by either phage alone is abortive and no progeny are made. The two genes are separated by over half the genome length [44]. Coinfections are therefore expected to generate recombinant, wild-type progeny, and the appearance of plaques can thus be used as proxy for coinfection.

**Figure 5:**
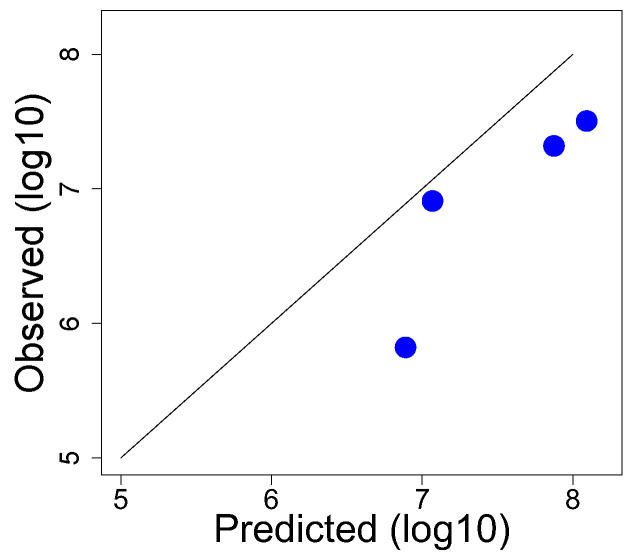
Comparison of observed and predicted coinfection levels using T7 deletion mutants. The *Y*-coordinate for each point (blue dot) is based on the plaque count of wild-type recombinants (a plaque requires recombination, which in turn requires coinfection). The *X*-coordinate contains post hoc calculations based on measured titers using 6 × 10^−9^ mL/min as the adsorption rate constant. The rate at which a coinfection generates a recombinant is not known but affects all predictions by the same magnitude. All points would lie on the diagonal if the predicted values exactly matched observed values. Data are in Table 2.

Under different conditions of cell or phage density, a known mixture of Δ*1* and Δ*16* was added to an *E. coli* K-12 host that supports growth of T7^+^ but not of either mutant. Dilutions of the culture were then plated to measure recombinants. Separately, using known phage titers and cell densities, and using an adsorption rate constant of 6 × 10^−9^ mL/min [45], expected coinfection levels were calculated numerically post hoc. A comparison of the two data sets reveals a broad correlation between observed and predicted, but it also reveals considerable discrepancies (Table 3, Figure 5).

A systematic deviation between prediction and observation is likely unavoidable. The probability of a coinfection yielding a wild-type recombinant phage that goes on to form a plaque is not known. Given the distance between the deletions, recombination frequency should be high [43], but there is no immediate way of knowing whether the probability is, for example, near 1.0 or closer to 0.2. Thus, the actual coinfection level exceeds that of plaque formation by an unknown amount, and the predicted values will then exceed observed values. This discrepancy would only have the effect of causing the points in Figure 5 to lie below the diagonal, which is observed. However, if that was the only source of error, the predicted values should fall on a line parallel to but below the diagonal on this log−log plot. That uniformity of deviation across points is not observed, suggesting other causes of error. Indeed, absolute recombination frequencies do vary in different experiments, although genetic order determinations are consistent [43].

In any system, the relevant sources of error can include the following:

(1) Variation in dilutions, plating conditions, the state of cells used for plating, and sampling error in plaque and cell counts will combine to generate errors in estimated titers. Note that a titer yielding a count of 400 plaques still yields a 2*σ* error of ±10% in estimating phage concentration.
(2) When different strains are used for measuring phage titers than for recombination, the efficiency of plating may differ among the strains, leading to a systematic error in the estimated force of infection.
(3) The time interval in which coinfection allows recombination will rarely be known.

Given these challenges, although coinfection levels will at best be predicted only approximately, even in well-characterized systems, those predictions remain useful in evaluating different protocols.

### 2.4. Recombinant Numbers

Coinfection is necessary for recombination, and, in a single-step protocol, maximizing coinfection should maximize recombinants; however, some protocols may include several rounds of renewed coinfection to allow rare recombinant genotypes to accumulate. In this situation the only practical transfer protocol is to use the lysate from one infected culture to inoculate the next culture. This may be conducted for several cycles without analyzing intermediate lysates before the next cycle (serial passage). In this scenario, the desired recombinants may occur not only from coinfections by the parental phages but also from coinfections between recombinants and parentals, or even between two recombinants. Furthermore, fitness differences among the phages can lead to changes in abundance of different phages, which in turn affects coinfection levels in subsequent rounds of coinfection. How does the incidence of recombinants change with these effects, and what modifications of a protocol might improve recombinant levels?

We apply a different numerical model to address basic questions of recombinant frequencies. This model assumes four loci with either of two alleles, as well as an infinite population size and deterministic recombination, which is a standard approach in models of population genetics [46]. There is no infection per se, just a population of individuals; recombinant frequencies are determined by first calculating frequencies of all possible genotype pairings, then calculating the frequencies of recombinant progeny within each pairing. With four loci, rates of recombination are specified among each of the three pairs of adjacent loci. For a single pair of parental genotypes, progeny genotypes are calculated for all eight possible recombination/non-recombination events across the four loci at the specified rates. However, not all genotypes will grow as well as others. This can be quantified by a parameter termed fitness, usually expressed as a fold-increase in numbers per unit time. Unequal fitness can be assigned as a simple multiplier of progeny genotype numbers.

The initial population consists of just the two parental types differing at all four loci; initial parental frequencies can be set to any level. Fitness effects are included to allow one parental type to be inferior to all other genotypes, as, when one parental phage type has a higher growth rate than the other. A single environment is assumed, representing growth on a permissive host. The model thus considers the accumulation of recombinants in the absence of selection for growth on a new (non-permissive) host.

#### 2.4.1. Model Basics

**Figure 6:**
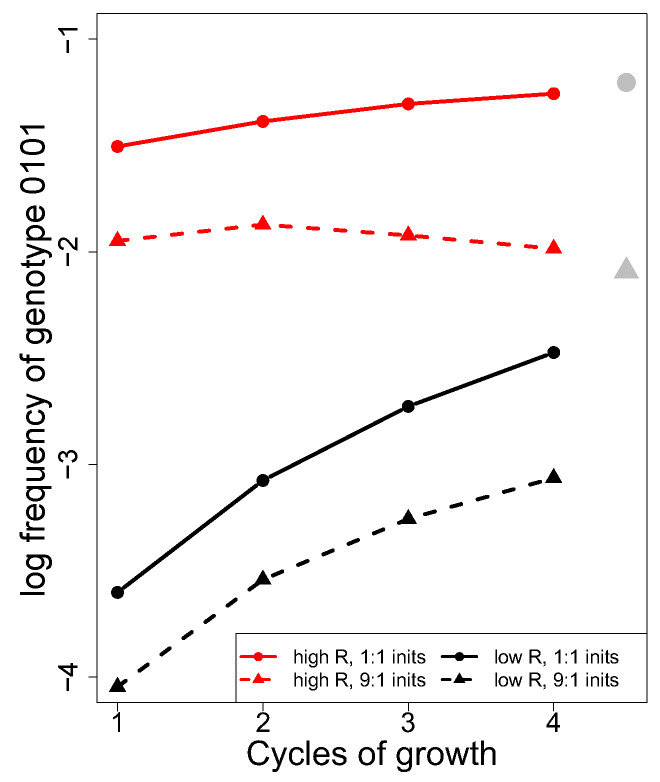
Deterministic recombinant levels depend on recombination rates across loci and on the relative initial abundance of parental genotypes. The *Y*-axis gives the (log_10_) frequency of the 0101 genotype when the parental genotypes are 0000 and 1111. Curves show 0101 levels over four generations; the long-term equilibrium recombinant level is given by the gray symbols at the right: the circle applies to the solid lines, the triangle to the dashed lines. Parental genotypes were equally abundant (solid) or in a ratio of 9:1 (dashed), thus affecting the final equilibrium. Recombination rates between the four loci were either all 0.5 (red) or all 0.1 (black), and all genotypes are assumed to have equal fitness. The C code is provided in Supplemental File S3_4locus_suppl_everyN.c.

At first, we only discuss a simple case where all genotypes have the same fitness (Figure 6). The trials in this figure vary in the three recombination rates between adjacent loci (0.5, 0.5, 0.5, shown in red; 0.1, 0.1, 0.1, shown in black, representing two extremes) and in the initial abundance of the two parentals (equal abundance for solid line, 9:1 for dashed). The parental phages have genotypes of 0000 and 1111, and data are plotted for the frequency of the 0101 genotype, which requires recombination between the two parental genotypes at all three sites (equally so for the complementary 1010 genotype). Note that this genotype can be created by fewer recombination events using pre-existing recombinants from an earlier cycle of growth. The 0101 genotype forms approximately 100-fold faster with high recombination rates than with low recombination rates, and equal abundance of the two parentals not only results in a higher initial frequency of 0101 but also a higher frequency at equilibrium (Figure 6). The gray circle at the right is the equilibrium value with equal parental abundance, while the gray triangle is the equilibrium for 9:1 relative abundance. None of this is surprising.

One potentially puzzling result, and one of possible relevance to phage infection protocols, is that, in one of the four trials, the frequency of the 0101 genotype is approximately constant over time (dashed red line). This experiment started with a 9:1 ratio of parentals and had maximal recombination. Such an outcome might suggest that there is no benefit to multiple rounds of recombination in obtaining a rare genotype. However, it can be shown that this outcome is a direct consequence of one parental genotype being introduced at low frequency. It is clear from Figure 6 that, at least when all genotypes have equal fitness on the permissive host, rare recombinants will be most abundant whenever equal parental frequencies are used.

#### 2.4.2. Unequal Parental Fitness (Models)

Different phages will often have different growth rates on the same host [47,48,49]; these differences can result in order-of-magnitude changes in relative phage abundances after a period of growth, even when grown in isolation and, in particular, when the different phages are grown together and compete for the same hosts. One consequence of fitness differences is that parental genotypes will not remain equally abundant over multiple rounds of coinfection. Is it possible to account for this possibility when designing a protocol to isolate a superior recombinant phage?

**Figure 7:**
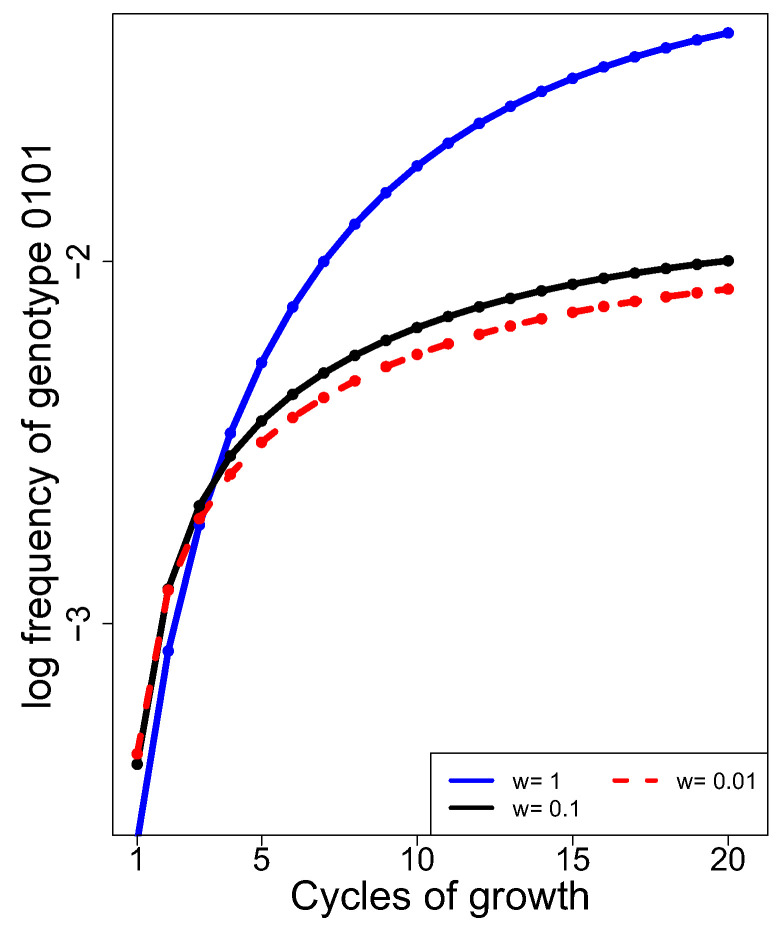
Unequal fitness of the two parental phages lowers recombinant accumulation over 20 cycles of growth (generations). Here, 15 of the 16 possible genotypes had equal fitness, whereas the 1111 genotype had lower fitness (black and dashed red curves) as per ‘w’ in the key inset. The case of equal fitness is provided for comparison (solid blue). Differences among the trials in the first 1–2 generations stem from specifics of the model and are not of importance.

From Figure 6, we might infer that unequal fitness will retard the accumulation of recombinants when more than one cycle of growth/generation is conducted, as unequal fitness will bias the abundances of the different genotypes. Recombinant accumulation curves were thus compared for the case of low recombination rates (0.1, 0.1, 0.1) between adjacent loci when the 1111 parental genotype has low fitness, relative to all other equally fit genotypes whose fitness is 1.0 (Figure 7). The long-term trend is clear: a fitness disparity between the two parental genotypes dampens recombinant accumulation. At 20 cycles, the difference between the heights of the equal fitness curve (blue) and the unequal fitness curve is 5-fold (dashed red, for a 100-fold difference in fitness) and 4-fold (black, for a 10-fold difference). On the log_10_ scale, these differences are, respectively, 0.7 (5-fold) and 0.6 (4-fold). The curves may be thought surprisingly similar for the cases where one parent has a 10-fold or a 100-fold lower fitness. However, the effect of a 90% reduction in relative fitness is already extreme, so reducing it to 99% will not have a profoundly greater effect.

**Figure 8:**
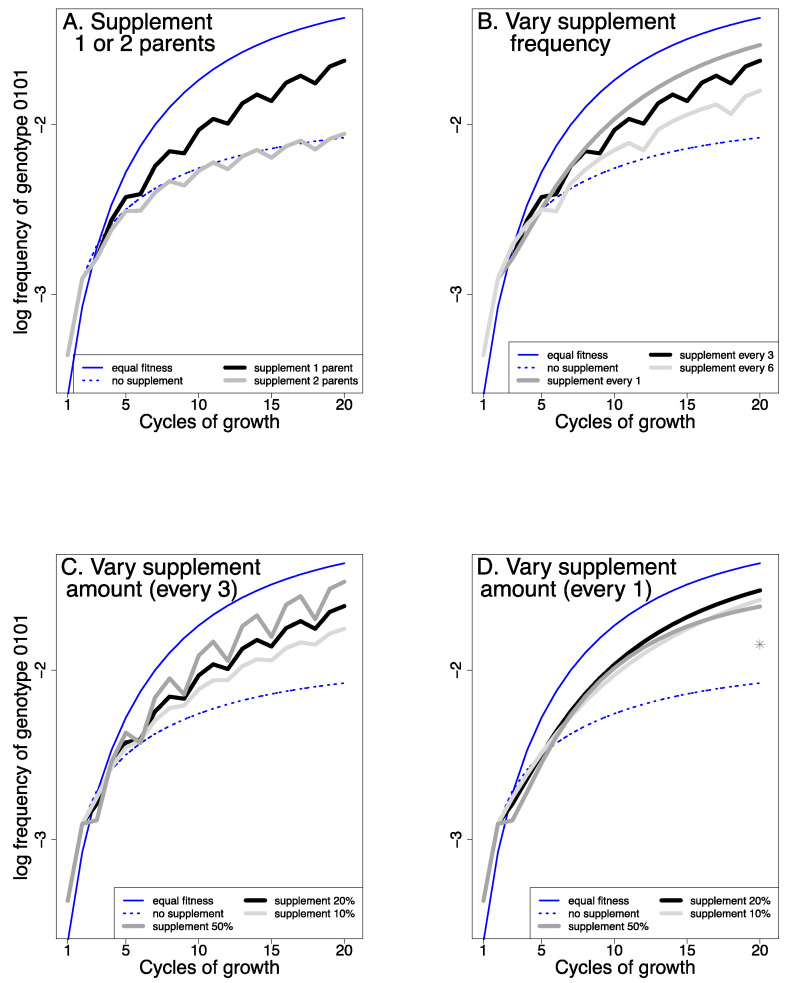
Supplementation boosts recombinant abundance over time (of genotype 0101). The dotted and solid thin blue curves are carried over from Figure 7 to show the effect of no supplementation (with and without a fitness difference). For all but the solid, thin blue curve (equal fitness), the fitness of the 1111 genotype = 0.01. (**A**) The black and gray curves show two types of supplementation when the 1111 genotype has fitness 0.01: supplementing just the 1111 parent (black) or supplementing both parents (gray). Supplementing with both parents offers no advantage, whereas supplementing with just the inferior genotype (1111) improves recombinant levels. The total supplementation level was 20% of the population, every 3rd generation. In (**B**–**D**), supplementation is restricted to the inferior genotype. (**B**) When holding supplementation to 20%, supplementing every cycle yields the highest level of recombinants. (**C**) When supplementing every third generation, supplementing with 50% is better than supplementing 20% or 10%. (**D**) When supplementing every generation, supplementing 50%, 20% and 10% have similar effects, but 20% is superior (beyond about Cycle 8). The gray star shows the effect at 20 generations of supplementing with 100%.

Fitness differences among phages cannot be directly corrected a priori with any standard protocol. However, some types of compensating adjustments may be practical, such as supplementing successive cultures with the parental phages to bring their abundances back towards equality [50]. For purposes of illustration, Figure 8 reveals the effects of different protocols of supplementation for the most extreme case of unequal fitness shown in Figure 7 (w = 0.01, where the low fitness of genotype 1111 results in a 5-fold reduction in recombinants [0.7 on the log_10_ scale]). For ease of interpretation, all Figure 8 panels show the no-supplementation curves from Figure 7 (blue is for equal fitness, red is for the 1111 parent having a fitness of 0.01 relative to that of other genotypes). Thus, the best possible outcome for supplementation would be to match the blue curve.

The trials reveal several important outcomes. First, supplementation of just the inferior parent performs far better than supplementation of both parents (panel A). Indeed, supplementation of both parents offers no benefit for the trial shown (contradicting the suggestion of [50]). Second, when cultures are supplemented intermittently (not every cycle), the curves show a jagged effect. This intermittent addition of parental genotypes allows us to disentangle two opposing forces. Specifically, supplementation is actually detrimental to recombinant frequencies when the phages are first added, which occurs because the addition of parental genomes reduces the frequencies of recombinants already in the population (e.g., if the phage population is doubled by adding parental genotypes, the frequencies of each recombinant will be halved) and is evident from the downward trajectories lasting a single cycle. The subsequent rebound in recombinant frequencies reflects the increased recombination from supplementation. The jaggedness of the curve does not occur when supplementation occurs every cycle, as these two effects are not separated in time. Third, at least in the simple system being modeled, supplementation every cycle may be superior to every third cycle. Fourth, there is a range in which supplementation is beneficial, and there may even be an optimal level (about 20% in this example). Adding 100% of the less-fit phage every cycle is predicted to be less effective (gray star in Figure 8, panel D), implying there may be “too much of a good thing”. However, the benefit of supplementation is realized over a broad range of levels, suggesting that protocols do not need to be finely tuned. At the same time, we caution that these analyses are preliminary, and the quantitative details should not yet be used to design protocols. At a minimum, further analysis is needed to address cases when some recombinant genotypes are inferior in fitness, as well as cases in which fitness differences are not so extreme.

When using uncharacterized phages, it may be difficult to know which parental phage—if any—is being lost. Genomic analyses of the phage pool or plating on phage-specific hosts would obviously allow such a determination, but either approach requires substantial time and effort. Given that supplementing with only the inferior parent appears to offer a benefit, the most useful easy protocol might be to conduct separate recombination lines, each being supplemented with a different parent (but only if an initial protocol not involving supplementation fails).

Perhaps the most extreme violation of practical phage protocols in this model involves calculating recombination rates in proportion to genotype frequencies. This assumption would represent simultaneous coinfection levels that exactly match phage genome frequencies, a virtual impossibility. Thus, the accumulation of recombinants in the model is much faster than would be obtained by experiment (as can be inferred from Figure 2, Figure 3 and Figure 4). Of course, the fourteen possible recombinant genotypes in this model comprise a vastly smaller number than the number of possible recombinants between any two phages. Indeed, when considering even a 10 kb genome, recombination could potentially occur at any base position, resulting in approximately 10^4^ possible single exchange points, 10^8^ possible double exchanges, 10^12^ triple exchanges, and so on. However, most of the potential recombinants between two phages will never occur, some will only occur after many generations, and many of those that do arise will be so rare as to be dominated by stochastic events. Any deterministic model with few genotypes, as employed here as a trivial example, is therefore best used only to identify qualitative behaviors and trends.

These trials are offered merely to reveal that there are indeed possible benefits to supplementation. They show that not all forms of supplementation are useful, and they give a sense of the robustness in some properties of a protocol. If supplementation appears to be both practical and useful, additional modeling will no doubt help identify the best protocol practices.

#### 2.4.3. An Experimental Study

The main generality evident from simple trials of the model is that recombinants accumulate over time, at least when recombinants are generated at a low rate per cycle. To obtain a sense of whether this theoretical generality is also an empirical generality, a second pair of phages was employed: phage T3^+^ and a recombinant T7 phage carrying the *E. coli* thioredoxin gene, T7/*trxA^+^*. Phages T3 and T7 are considered close relatives; their genomes are syntenic, and many genes of one phage complement defects in the other phage [51]. Both T3^+^ and T7^+^ require the host thioredoxin for DNA replication, but T7 derivatives encoding *E. coli trxA^+^* (T7/*trxA^+^*) grow on *trxA* mutant hosts. TrxA acts *in trans* and thus in a *trxA* mutant cell, while co-infection by T7/*trxA^+^* and T3^+^ will allow the latter to replicate. Two exceptions to the generality of heterologous complementation are pertinent here: T7 and T3 RNA polymerase (gene *1* product) do not recognize the heterologous promoter, and, whereas T3 gene *1.2* is essential for phage growth on F plasmid-containing strains, T7 gene *1.2* is one cause of an abortive infection on those strains [52]. T7 gene *1.2* is recessive to T3 gene *1.2*, and thus co-infection allows growth of both phages in F strains. A *trxA^-^* strain carrying the F plasmid (IJ2405) is thus non-permissive for T7/*trxA^+^* and T3^+^ individually but will support growth of both phages in a co-infection or, obviously, by single infection of a recombinant harboring both T3 gene *1.2* and *trxA^+^* [51].

There are two obvious paths by which recombination can create a phage that will grow on the non-permissive host IJ2405. One moves *trxA^+^* from T7 into T3; the other moves T3 gene *1.2* into T7/*trxA^+^*. It would seem that these recombinations should be simple and easily obtained, although biological reasons why this is not necessarily so have been discussed [52]. The issue here is not so much the ease or difficulty of obtaining the recombinants; rather, the issue is one of recombinant accumulation over time and the repeatability of the outcome.

A mixture of T7/*trxA^+^* and T3^+^ was added to a permissive host at a high MOI (~2 for each phage) and a presumed high FOI, with the culture being grown to lysis. A high FOI for T7 (~6) was known from its high titer and a known adsorption rate, but the FOI for T3^+^ was not known because the adsorption rate had not been measured. The lysate was promptly added to a second culture and grown to lysis, and the cycle was repeated a third time. The entire protocol was independently replicated, yielding two accumulation curves.

While the MOI of the first culture was known approximately in advance (based on optical density of the cell culture and phage titers), the titers of the second and third cultures and all cell densities were measured by plating on IJ511 after the fact, then using selective hosts to estimate the proportions of each parental phage (see Section 4; all exceeded 2.0 for both phages). Assuming that the adsorption rate constants of T3 and T7 are the same, all FOI values were close to or exceeded five. These conditions mimic those modeled in the upper right corners of Figure 2, Figure 3 and Figure 4.

**Figure 9:**
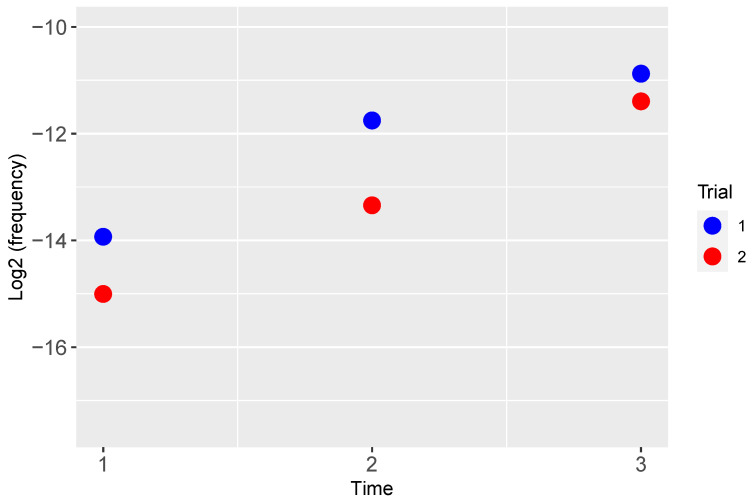
Frequency of recombinants over time between T3^+^ × T7/*trxA^+^*, whose genomes now contain T3 gene *1.2* and the T7-derived *trxA^+^*, during serial transfer on a permissive host. Two independent trials are illustrated. Time is the cycle of phage growth in successive transfers.

Densities of recombinants containing T3 gene *1.2* and *trxA^+^* were measured as a fraction of the total phage population (Figure 9). Both trials show a monotonic increase in recombinant frequency. Note that there was no known selection for recombinants during growth of the phage mixture: both T7*/trxA^+^* and T3^+^ reproduce normally on the permissive host, hence the growth advantage of a recombinant is only manifest on the selective host used (IJ2405). Each point is a single sample, hence without error estimates, but, by a non-parametric permutations test, the monotonic increase in the two independent trials is significant at *p* < 0.028. The initial recombinant frequencies lie between 10^−4^ and 10^−5^ (illustrated on a log_2_ scale to facilitate comparing the differences), differing only about 2-fold in the two trials. These empirical measures are broadly consistent with the model: recombinant frequencies increase with the number of cycles of growth. The result justifies protocols that sequentially provide opportunities for recombination. Current technology would allow for a much greater depth of analysis, particularly with sequencing being utilized to measure the incidence of recombinant DNA molecules. However, the industrial or most useful goal is to describe a simple, broadly applicable protocol that will enhance recombination among diverse, uncharacterized phages. Ensuring high levels of coinfection combined with sequential propagation of recombinant mixes is a significant step to achieving that end.

## 3. Discussion

This paper has modeled several aspects of the control of phage coinfection toward the goal of recombination, supported by a few empirical efforts with well-characterized phages. The models explored ways to maximize coinfection levels in the initial mix of phage and bacteria as well as methods to maximize ‘rare’ recombinants over multiple rounds of infection when one parental phage has lower fitness than the other.

The precise, empirical control of recombination levels with uncharacterized phages is not practical. It requires detailed knowledge of phage life histories as well as measurements of adsorption rate coefficients on the common hosts. The empirical studies qualitatively supported the models but also illustrated the difficulty of controlling coinfection levels tightly, even in well-characterized systems. The difficulty results largely from the sensitivity of coinfection levels to measures of titers and adsorption rate constants. Better coinfection levels could no doubt be attained by using higher phage concentrations, but that may only be realized by using concentrated stocks, not simple lysates. Attempts to control recombination rigorously with wild, uncharacterized phages will thus be even more challenging.

The modeling efforts have nonetheless revealed some results of practical utility that may not be instinctively obvious. For example, use of high MOIs of the different phages does not ensure high levels of coinfection in a short window of time; high FOIs of the different phages do, albeit provided that the MOIs are at least 1.0. However, measuring the FOI requires knowing both phage titers and adsorption rates, which is achievable but requires time-consuming initial characterization. As another example, different fitnesses of the parental phages hamper the accumulation of recombinants beyond the first cycle of growth. Supplementing sequential recombinant mixes can recover some of this ‘lost’ recombination, but only if the supplementation is of just the inferior parental type and not if the supplementation is of both parental types. Some of us had casually suggested that supplementing with all parental types would be beneficial [50]; that suggestion is overturned by results presented here and shows the benefit of modeling over intuition. Our modeling also suggests that, although the quantitative benefit of supplementation depends on protocol details, such as amount and timing, a qualitative benefit is robust to many details. Since it may be difficult to determine which, if any, parent has inferior fitness, the solution may be to attempt separate protocols in which supplementation is limited to one of the parents.

Despite the difficulty of accurately predicting and controlling recombinant levels with mixed phage types, we suggest that recombination is a worthy objective in evolving novel host ranges. Mutation alone may often not be sufficient [22]. The alternative approach of genetically engineering an expanded host range [24,27,41,53] may be better than relying on mutation, but it has the disadvantages of requiring (i) substantial infrastructure specific to each phage and (ii) knowledge of the genes that might contribute to host range evolution. Use of natural recombination is intrinsically blind to such prior knowledge and requires no infrastructure. One message from this study is that it will be difficult to know or to precisely control coinfection/recombination levels in any specific implementation. Nonetheless, high levels of coinfection can be attained merely by ensuring that a protocol encompasses a high force of infection and at least moderately high multiplicity for the different phages. Toward this end, protocols such as Appelmans may enable adequate levels of recombination without formal measures of phage titer and cell concentrations [22,54]. However, potentially subtle differences in Appelmans-like protocols could have major effects on recombination, such as whether the phage pool from each round includes or excludes the high-FOI cultures (compare [22,25] and [54]).

The models here are limited to a potentially narrow range of conditions, and additional studies are warranted to address other possibilities. (1) For some phages, recombination may occur between an infecting phage and prophages in the bacterial genome. This process would allow recombination of a single infecting type at any MOI, but it relies on the infecting phage not degrading the host DNA. (2) The time interval in which coinfection results in recombination might be subject to experimental control of the bacterium, such as by starvation or use of low temperature. Increasing this interval would reduce the requirement of a high force of infection but still require a high MOI.

The paper has addressed the accumulation of recombinants in the absence of any known selection for those recombinants, as when the cultures use only the fully permissive host. It is of course feasible to conduct protocols in this way, periodically plating on the non-permissive host to search for plaques while continuing recombination efforts using just the permissive host. However, the odds of getting a suitable recombinant without selection will often be infinitesimal, as the number of possible recombinants will vastly exceed the numbers of phages that can be contained in any feasible culture. Without selection for intermediates, a recombinant with the desired properties will often be unattainable. Note that the empirical tests used here were designed so that recombination events allowing growth on the non-permissive host were especially simple, thus making them non-representative of the usual challenges faced when trying to change host range.

Phage recombination during treatment of a patient is presumably unavoidable. Even use of a single treatment phage need not prevent recombination over prophages in the bacterial genome. However, recombination leading to a functional change in phage progeny—evolution of a new phage type—is presumably a low-probability event, and, as treatment phages are deliberately chosen to infect the pathogen, there is presumably little selection for phages to evolve new host ranges during treatment.

## 4. Materials and Methods

### 4.1. Phage and Bacterial Strains

T7 4107 [55], here called T7∆1, was propagated on BL21(DE3) [56,57], denoted IJ492 in Table 4. T7 ∆16 [58] was propagated on strains containing a plasmid-borne gene *16* [59]. T7/trxA^+^ has *E. coli trxA^+^* cloned into the gene *3.8* Bgl II site. All phages and bacterial strains (Table 4) are from the IJM collection; some strains listed were not used directly for assays here but were used in the construction of strains that were used here. Both bacteria and phages were propagated aerobically at 37 °C in LB broth (per liter: 10 g Bacto tryptone, 5 g yeast extract, 10 g NaCl). The solid media was LB broth containing 15 g Bacto Agar per liter or 7 g for top agar.

Coinfection assays of T7Δ1 and T7Δ16 were carried out by adding a mixture of both phages at known concentrations to exponentially grown IJ511 (~2 × 10^8^ cells/mL) for 2–4 min and plating aliquots for infective centers using uninfected IJ511 as carrier. As neither phage alone produces progeny on IJ511, plaque formation must be a result of coinfection followed by recombination to create wild-type phages. Plaque numbers were therefore assumed to be proportional to the number of coinfections.

Recombinants between T7/*trxA^+^* and T3^+^ were obtained by coinfecting a mixture of both phages to 1 mL of IJ511 using an overall MOI of ~2 for each phage. IJ511 is permissive to both phages, and after culture lysis, an aliquot was then added to a second culture. A third lysate was then obtained in the same way, and all three lysates were then plated on a permissive host (IJ511 or IJ1517) to obtain total phage titers and on IJ2405 or IJ2520 (F′*lac, trxA^−^*) to quantify large-plaque recombinants. Recombinants were found at 4–5 orders of magnitude lower than non-recombinants. After the fact, it was determined that the MOI of all subsequent cultures was also at least 2. IJ512K and IJ1748 were used to estimate relative amounts of T7/*trxA^+^* and T3^+^ in the cultures so that a known total titer could be partitioned into titers of each phage.

### 4.2. Models of Coinfection

The coinfection model used for Figure 2, Figure 3 and Figure 4 was given above in equation set (1). It was implemented in C using the Euler method with a step size of 0.01 (Supplement File S1). To model predicted coinfection levels for Figure 5, a C-program was written (Supplement File S2) that enforced a strict 2 min period for coinfection of individual cells, a 3 min mix of cells and phage before plating, and an adsorption rate constant of 6.1 × 10^−9^ [45]. The inputs were the empirically measured cell and phage densities for the trial.

### 4.3. Models of Recombination

The deterministic model of recombination among 4 bi-allelic loci (Supplement File S3) was encoded in C as a set of discrete-time difference equations. It specified frequencies of all 16 possible genotypes, calculated the frequencies of all possible 16 × 16 genotype pairings and then exhaustively summed the contribution of the pairing to all 16 possible progeny genotypes based on recombination frequencies and parental alleles. Fitness was assigned (after recombination) as a multiplier of the genotype frequency. Genotype frequencies were normalized to 1 at the end of each cycle.

## 5. Conclusions

Recombination among heterologous phages may augment the evolution of new host ranges. Furthermore, recombination may occur in the experimental propagation of phages and can be substantially influenced by the protocols used in that propagation. The first step in enabling recombination is to rapidly coinfect permissive cells by the different phages. Although coinfection can be controlled experimentally, it is not intuitively obvious how best to achieve high levels. Computational models showed that use of a high MOI (multiplicity of infection) for both phages is not sufficient; one must also ensure a high force of infection (FOI) of both phages, which requires a high titer of the phages. Even so, subtle effects accrue to the balance of cell densities, FOI, and MOI when optimizing coinfections. Empirically, however, it proved difficult to attain tight experimental control of coinfection levels.

A single round of experimental coinfection/recombination will rarely be sufficient to obtain an abundant diversity of recombinants for the rapid evolution of host range. We showed both experimentally and computationally that successive rounds of coinfection will boost the accumulation of recombinants. However, serial passaging raises a new problem when one parent is less fit, and ongoing propagation will reduce its abundance and thus reduce recombination. Computational models showed that recombinant levels can be largely restored if successive cultures are supplemented with the less-fit parent exclusively.

## Figures and Tables

**Table 1:** Variables and parameters used for coinfection model (1).

Terms	Meaning	Initial Values Used
Variables (all functions of time)		
*B*	Density of uninfected bacteria (/mL)	10^8^, 10^7^, 10^6^
*P_i_*	Density of phage *i* (*i* = 1, 2) (/mL)	Given in Figures
*X_ij_*	Density of cell infected with phage *i* (/mL) (*i* = 1, 2) *j* = *T* for temporary, *P* for permanent *ij* = 12 indicates coinfection	0
Parameters		
*k*	Adsorption rate constant (mL/min)	10^−9^
*δ*	Rate constant of escape from coinfection (/min)	0.5
*r*	Bacterial growth rate (/min)	

**Table 2:** Relationship between phage density and FOI.

**Phage density** (/mL)	10^6^	10^7^	10^8^	10^9^
**FOI (/min)**				
(*k* = 10^−8^ mL/min)	0.01	0.1	1	10
(*k* = 10^−9^ mL/min)	0.001	0.01	0.1	1
(*k* = 10^−10^ mL/min)	0.0001	0.001	0.01	0.1

**Table 3:** Data used to compare predicted and observed coinfection levels.

Δ1 Density	Δ16 Density	Cell Density	Predicted	Observed
1.475 × 10^8^	3.234 × 10^8^	1.5 × 10^8^	7.5 × 10^7^	2.1 × 10^7^
2.95 × 10^8^	6.469 × 10^8^	1.55 × 10^8^	1.24 × 10^8^	3.2 × 10^7^
2.95 × 10^7^	6.469 × 10^7^	1.55 × 10^8^	7.75 × 10^6^	6.636 × 10^5^
2.95 × 10^8^	6.469 × 10^8^	1.2 × 10^7^	1.17 × 10^7^	8.2 × 10^6^

**Table 4:** Bacterial strains (*E. coli*).

Strain	Genotype
IJ511	K-12 ∆*lacX74 supE44 galK2 galT22 mcrA rfbD1 mcrB1 hsdS3*
IJ512K	IJ511/F′*lac*-Kn^R^
IJ1517	K-12 ∆*lacX74 thi* ∆(*mcrC-mrr)* Tc^s^
IJ1748	IJ1517 *trxA*::Kn
IJ2405	IJ1517 *trxA*::Kn/F’*lac*-Tc^R^
IJ492	[BL21(DE3)] Expresses T7 gene *1* (RNAP)
IJ493	[BL21] *E. coli* B *lon*::IS*186* Δ(*galM-ybhJ*) *ompT hsdS* (λ^S^)

## Data Availability

Data generated by computational analyses may be regenerated using the software code provided in the Supplementary Materials. The empirical data are provided in Figure 5 and Figure 9 and Table 3.

## Supplementary Materials

The following supporting information can be downloaded at: https://www.mdpi.com/article/10.3390/cells13070585/s1, File S1_coinfection.c: The coinfection model used for Figure 2, Figure 3 and Figure 4, using equation set (1) implemented in C using the Euler method with a step size of 0.01. File S2_delay_coinfection.c: A C-program to predict coinfection levels for Figure 5, enforcing a strict 2 min period for coinfection of individual cells, calculated for a 3 min mix of cells and phages before plating and an adsorption rate constant of 6.1 × 10^−9^ mL/min [45]. Inputs were the empirically measured cell and phage densities for the trial, given in Table 3. File S3_4locus_suppl_everyN.c: The deterministic model of recombination among 4 bi-allelic loci implemented in C as a set of discrete-time difference equations.

## Appendix A. Mathematics of Why MOI Fails to Provide Coinfection Levels

The most confusing aspects of Figure 2, Figure 3 and Figure 4 may stem from the virtual irrelevance of the MOI to the statistics of interest. To help make it more intuitive, we provide a simple ‘balls in bins’ model. Suppose there are *N* bins and *P* balls; ‘bins’ are bacteria, ‘balls’ are phages. To represent infection, each ball (phage) is randomly assigned to a bin (bacterium) with the probability 1/*N* for each bin regardless of whether the bin is already occupied (infected). After all *P* balls have been assigned to bins (all phages have gone on to infect), the probability that a given bin is empty (i.e., the cell remains uninfected) is merely the chance that it ‘dodged the bullet’ from all the *P* balls (phages):

(A1) $${\left(\frac{N-1}{N}\right)}^{P}={\left(1-\frac{1}{N}\right)}^{P}.$$

The above expression can be converted into something more useful. For mathematical convenience, we can rewrite *P = c N*, where *c* = *MOI*. If *N* is large (e.g., thousands, whereby specific approximations are valid), it is well established that the above probability of a bin being empty is approximately $e^{-c}$ and the expected *fraction* of uninfected cells is

(A2) $$e^{-MOI}.$$

This assumes that there is no limit on the amount of time for a phage to infect cells (the FOI is irrelevant here, because it is a rate measure). The reason that some cells remain uninfected when MOI > 1 is that other cells receive more than one phage.

The fraction of infected cells is thus 1 − $e^{-MOI}$. With two phage types, assuming no coinfection interference, the fraction of cells coinfected is

(A3) $$\left(1-e^{-MOI_{1}}\right)\left(1-e^{-MOI_{2}}\right),$$

with $MOI_{1}=\;P_{1}/N$ and $MOI_{2}=\;P_{2}/N$. Again, this assumes an unlimited time for the infection. The expected *number* of coinfections is simply *N* times this fraction. Thus, if $MOI_{i}=1$ for both phage types, $(1-e^{-1})=0.63$, and so ~40% will ultimately be coinfected.

To accommodate a limited time for infection, *T*, note that the per capita adsorption rate for a phage is *kN* (where *k* is the adsorption rate constant above and assuming that a cell remains open to repeated infections during the entire incubation time). Note that *kN* is a ‘reverse-perspective’ FOI, meaning that it gives the rate at which an individual phage is adsorbed by a cell, whereas the FOI is the rate at which an individual cell is infected by a phage. Thus, the fraction of phages that do not adsorb in time *T* is $e^{-kNT}$, and these phages are essentially wasted. With this time constraint, the above fraction of coinfected cells requires replacing $MOI_{i}$ with the “effective MOI”.

(A4) $$moi_{i}=\frac{P_{i}}{N}(1-e^{-kNT}).$$

Finally, if we want to account for singly infected cells becoming ‘protected’ against coinfection at rate *δ*, the equation for the fraction of coinfected cells is corrected with a simple multiplicative term,

(A5) $$\left(1-e^{-moi_{1}}\right)\left(1-e^{-moi_{2}}\right)\left(\frac{kN}{kN+\delta }\right).$$

This final result now elucidates three causes for the failure of coinfection levels to reach the level that would be suggested by the MOI: the random distribution of phage leaves some cells uninfected, the limited time for infection can leave even more uninfected cells, and the limited time window allowed for coinfection means that, although some cells may eventually receive both phages, they do not qualify as coinfected for the purposes of recombination.
